# Erratum to: *Genes and Environment*, Vol. 39

**DOI:** 10.1186/s41021-017-0080-1

**Published:** 2017-06-02

**Authors:** Genes and Environment

**Affiliations:** London, UK

An error occurred during the publication of a number of articles in *Genes and Environment*. These articles were erroneously published in volume number 39, which is listed with a publication date of 2017. However, these articles were published in final form in the year 2016.

Provided below are the actual publication dates and the correct citation for each affected article. Please note that the citation refers to volume number 39 with the year as 2017.

Article: http://dx.doi.org/10.1186/s41021-016-0048-6

Publication date: 22 June 2016

Correct citation: Yagi, T. Genes and Environ (2017) 39: 1. doi:10.1186/s41021-016-0048-6

Article: http://dx.doi.org/10.1186/s41021-016-0051-y

Publication date: 1 December 2016

Correct citation: Suzuki, T. & Kamiya, H. Genes and Environ (2017) 39: 2. doi:10.1186/s41021-016-0051-y

Since this error was detected the journal resumed publication in the 2016 volume, volume number 38, for the remainder of that year. In 2017, the journal resumed publication in volume number 39. The publisher apologizes for any confusion caused by this error.

